# Online Correction of Laser Head Nozzle Position for Laser Metal Deposition Using a Chromatic Confocal Displacement System

**DOI:** 10.3390/s23167120

**Published:** 2023-08-11

**Authors:** Piotr Koruba, Grzegorz Iskierka, Bartosz Poskart, Jakub Mazur, Adrian Zakrzewski

**Affiliations:** Department of Laser Technologies, Automation and Production Engineering, Wroclaw University of Science and Technology (WUST), Wyb. Wyspianskiego 27, 50-370 Wroclaw, Poland; grzegorz.iskierka@pwr.edu.pl (G.I.); bartosz.poskart@pwr.edu.pl (B.P.); jakub.mazur@pwr.edu.pl (J.M.); adrian.zakrzewski@pwr.edu.pl (A.Z.)

**Keywords:** confocal displacement sensor, laser metal deposition, digital image correlation, laser material processing

## Abstract

The stability and repeatability of laser metal deposition is particularly important when processing multiple layers or depositing material on complex component surfaces, and requires the use of process parameter control including the stand-off distance between the laser head and the substrate. The system proposed in this paper for correcting the stand-off parameter is based on a chromatic confocal sensor integrated into a laser head. Then, the spectral signal acquired from the measurement system is processed by using the developed application to compensate for the movement of an additional axis of the kinematic system. This study used an independent verification system based on the digital image correlation method. The validation tests were carried out using the system for correcting the stand-off parameter with different control algorithms and given motion trajectories and substrate materials. The results demonstrate that the developed system can be useful for laser metal deposition.

## 1. Introduction

Additive manufacturing and remanufacturing of parts are becoming increasingly common techniques due to material savings, shorter delivery times, and environmental concerns based on minimizing emissions [1]. When using direct energy deposition (DED) techniques such as laser beams in the laser metal deposition (LMD) process, it is also possible to manufacture parts with larger dimensions and to achieve higher material deposition rates [2]. The development of laser metal deposition technology for more complex geometries requires an advanced system for control of process parameters or quantities correlated with them such as melt pool temperature [3], dimensions [4], or clad geometry [5]. The use of a process control system is essential for achieving process repeatability in additive manufacturing/remanufacturing of complex parts. The process parameters that affect the qualitative properties of a deposited structure’s geometry are particularly important, and they are impossible or very costly to improve in post-processing or in a hybrid process. One such crucial parameter for the LMD process is the stand-off parameter defined as the distance between the laser head nozzle and the surface of the substrate material [2]. It is critical in the case of multilayer deposition, since it is closely linked with the thickness of a single clad layer as well as the width of a single clad [6].

In addition, the stand-off parameter affects the geometrical properties of a deposited structure, as well as its metallurgical, mechanical, and quality parameters. For example, the ratio of laser power to stand-off parameter influences surface roughness, microhardness, porosity, and deposition efficiency [7].

The effect of the stand-off parameter on catchment efficiency, which, in turn, significantly affected the height of a deposited structure, was presented by Donadello et al. in [8]. The authors also presented three operational ranges related to this parameter that could occur during the process of multilayer deposition: the self-regulation, nominal, and instability zones. The authors pointed out the possibility of using a closed-loop system to adjust the stand-off parameter during the process, especially in the case of loss of material deposition stability. However, in [9], it was not recommended to reposition the substrate or the laser head by changing the stand-off parameter between every layer due to insignificant changes in the thermodynamic conditions of the melt pool.

The presence of a self-regulation zone was also reported by Zhu et al., in [10], along with the dependence of the height of a single deposited layer when changing the stand-off parameter. The clad height reached a maximum value with a stand-off parameter value equal to the powder focus length.

Controlling the height of a single clad, and therefore the value of the stand-off parameter, were also the subject of research presented in [11], in which the authors demonstrated a control system based on a digital camera and field of view compensation methods as well as image processing through filtering and contour extraction. An extension of the described solution is the system proposed by Thiele et al. [12], where an infrared camera was used, which additionally permitted determination of the melt pool temperature. However, this method was sensitive to noise during the surfacing process and it was limited to thin-walled parts with less complexity due to the necessary side camera access. Another approach for controlling layer thickness during the LMD process was based on scanning of the consecutive layers with a structured light scanner, which allowed the manufactured component to be more consistent with the computer model, thus reducing post-process machining [13]. However, such a solution introduced additional time delays during the process, significantly increasing the part processing time. Furthermore, measuring the distance behind the laser head using a laser displacement sensor was proposed in [14], which involved a time delay and the need for additional kinematic system movements. Measuring the thickness of the deposited layer using laser displacement sensors during remanufacturing using the LMD process was also presented by Lu et al. as a single directional measurement [15].

The height of deposited material can also be estimated from the position of the melt pool, calculated based on the images acquired using an off-axis mounted camera [16], although this method is highly sensitive to the quality of the surfacing and has a rather large error in the transition areas.

Monitoring the height of the laser deposited layer has also been carried out using a laser triangulation system [17]. The developed measurement system was used to determine the deviation between the programmed trajectory of a robot and the coaxially measured layer height [18]. However, the authors pointed out that in order to achieve full functionality of the control system, it was necessary to synchronize it with the controller of the drives of the robot that performed movements in the laser deposition process. Another triangulation system for height measurement in directed energy deposition processes, including the LMD process, was also demonstrated by Borovkov et al. [19]. The developed system exhibited high robustness using a shape-to-shadow technique, even in the arc-based process. However, it provided a solution that was limited to single-axis deposition due to the lack of concentricity.

A multidirectional sensor system based on the laser triangulation method was also presented by Jothi Prakash et al. [20]. It allowed for clad geometry scanning during the process and simple visualization of the LMD process results, yet the sensor system was mounted on the side of the processing head, relatively close to the laser beam interaction zone, and its robust shielding lowered the flexibility and customization of this solution.

In this paper, a system was developed for online compensation of deviations in the the value of the stand-off parameter during the laser metal deposition process due to changes in substrate geometry or incorrectly designed trajectory of the laser head deposition nozzle. First, a preliminary study on the effect of changes in the value of the stand-off parameter on the results of the LMD process was conducted to quantify the requirements for the developed system. Subsequently, a measurement system based on a chromatic confocal sensor was proposed, which was integrated with a laser head for executing the deposition process. Compensation of the value of the stand-off parameter was realized by implementing an actuator in the form of a linear stage with increased positioning precision. The developed system was characterized in an open- and closed-loop control system.

In the presence of a feedback loop as a signal from a chromatic confocal sensor, the system’s operation was tested on a silver mirror and a low-alloy steel substrate. This allowed the system to be validated under operating conditions without an active laser beam.

## 2. Materials and Methods

Research on a system for correction of the stand-off parameter in the LMD process was carried out on a standard workstation for executing laser metal deposition with powder. The workstation to perform the LMD process consisted of a six-axis robot (REIS, RV 60-40, Germany), a laser generator (laserline, LDF 4000-30, Mülheim-Kärlich, Germany), and a powder feeding system (GTV PF 2/2). The low-alloy steel AISI 4330 (substrate) and the self-fluxing Ni-based alloy Metco 15F (powder) were selected as materials for the LMD process tests.

In the preliminary study on the influence of the value of the stand-off parameter, the use of a typical nozzle was used for the LMD process in the COAXpowerline laser head (Fraunfhofer, IWS, Dresden, Germany), designated as AA13. The LMD process was carried out for a wide range of stand-off parameter values, starting from 3 mm and ending at 33 mm. It is worth highlighting that the AA13 deposition nozzle used in the research has a nominal stand-off parameter value that is equal to 13 mm, which is within the proposed range. The remaining parameters of the LMD process are listed in Table 1.

### 2.1. Description of the Laser Head Integrated Chromatic Confocal System

**Figure 1:**
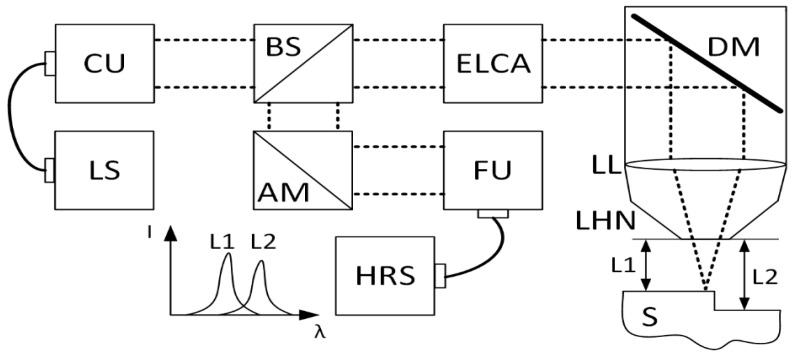
Schematic diagram of the LICCS system optical path.

In order to determine the distance (L) between the laser head nozzle (LHN) and the substrate material (S), an optical system developed by the authors was used (Figure 1). Its detailed description, in terms of operating principle, parameters, and functionality, has been presented in previous publications [21,22]. In brief, the optical system is a modified version of the chromatic confocal sensor (CCS), which is commonly used as a stand-alone displacement sensor. The modification involves its integration with laser head optics, which shapes the laser beam for application in the laser metal deposition process. As a consequence, the optical path of the laser beam is shared with the optical path of the system in the laser head part, forming the laser head integrated chromatic confocal system (LICCS). The principle of the optical system operation is based on the phenomenon of longitudinal chromatic aberration (LCA). As a result of this phenomenon, a spectral splitting of focal lengths occurs, i.e., each wavelength focuses on a different location in space along the optical axis of the system.

This focal splitting directly defines the measurement range of the system. If there is a material surface present within this measurement range, then only a single wavelength from the focal split range is focused on it. As a result, a characteristic spectral peak is observed on an optical spectrum analyzer, which is usually a spectrometer. Its amplitude argument corresponds to the wavelength focused on the surface of the material. By using the system’s calibration curve, defined as the dependence of the amplitude argument (wavelength) as a function of the material surface displacement, it is possible to determine relative distances with micrometric accuracy [23]. However, in the case of engineering material surfaces with significant roughness, the accuracy of such a system decreases [24].

In terms of the operating principles of the optical system, the measurement beam is generated from a broad-spectrum light source (LS) which is a single LED and coupled to the input optical fiber using the butt-coupling technique. The LS generates radiation in the 500–650 nm range. The core diameter of the input optical fiber is 50 µm with 0.22 NA. Subsequently, using a collimating unit (CU), the measurement beam is collimated and directed to a 50:50 non-polarizing plate beam splitter (BS). The transmission part of the measurement beam is directed to the enhanced LCA (ELCA) unit, where the focal lengths are spatially split, and then the measurement beam is re-collimated.

Due to the spatial splitting of the focal lengths, only one wavelength in the 500–650 nm range is well collimated behind the ELCA unit. It is 575 nm, and it is the central wavelength of the designed optical system. Other wavelengths are gently converging (shorter wavelengths) and diverging (longer wavelengths). Such a spectrally defined measurement beam is directed to the optical components of the laser head; it is reflected from the dichroic filter (DM) at an angle of 90 degrees to the optical axis of the path and it is focused on the surface S by a process lens (LL). After reflection from the surface S, the measurement beam is directed along the same optical path as before to the BS. After reflection from the BS, the measurement beam is propagated to an adjustable angle mirror (AM), and then to a focusing unit (FU) that focuses the beam on the face of an output optical fiber. The purpose of using an AM is to compensate for the non-zero thickness of the BS, which results in a slight shift in the propagation of the measurement beam with respect to the optical axis of the path. The output optical fiber is connected to a high-resolution spectrometer (HRS), on which a characteristic spectral peak is detected. All optical components used for the designed LICCS system are aspherical and achromatic with two exceptions: the LL, which is a PCX spherical lens, and the first lens in the ELCA unit which is a hyperchromatic lens. The developed LICCS system has an accuracy of 0.035 mm for the reference material (silver mirror) determined in accordance with the standard [25]. The accuracy value consists of trueness (the maximal difference between the measured sample position and the value derived from the calibration curve) and precision components (standard deviation of the error for approaching 0 mm displacement point) [21]. However, for the steel substrate, the average accuracy obtained is much higher, i.e., 0.09 mm and it is dependent on the surface quality of the sample. Multiple calculations of the accuracy parameter at different locations on the steel sample show that the range of this value is 0.06 mm in contrast to the reference, where it was only 0.004 mm.

### 2.2. System for Compensation of the Stand-Off Parameter Changing in the Laser Metal Deposition Process

**Figure 2:**
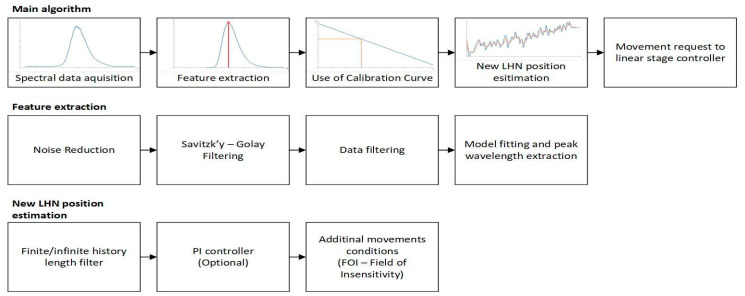
Block diagram of the implemented LMD-PCS control algorithms in the LabView-based application.

The LICCS system is designed to be used in laser material processing, particularly during the LMD process, during which the height of the deposited layer may vary and should be adjusted to avoid uneven surfaces, geometry warping, and other defects. Compensation of the height of the laser head nozzle during the process is done through an additional controllable axis (Standa, 8MT165-200-B43, Vilnius, Lithuania) mounted on a six-axis industrial robot. Functionality of the implemented LMD process compensation system (LMD-PCS) was complemented by the development of a LabView-based application that controlled the linear stage used as an additional axis through the utilization of a feedback loop from the designed LICCS system. A block diagram depicting the algorithms included in the application is shown in Figure 2.

The implemented control algorithm for the LMD process compensation system uses estimation of displacement between the LHN and the surface S based on signal generated by the LICCS to control the additional axis in a way that keeps the stand-off parameter constant. Calculation of the displacement was performed using a linear calibration curve that characterizes the relationship between the peak wavelength extracted from the LICCS signal and the LHN position. This method can be applied only within the sensor’s correct operating range, i.e., ±2 mm. The process of extracting the peak wavelength was realized in several stages as follows: Initially, the correctness of the obtained measurement is verified based on the minimum intensity and maximum full width at half maximum (FWHM) conditions, after which, if the assumptions are met, data reduction to the 400–800 nm range is performed, followed by dark current reduction. Then, a third order Savitzky–Golay filter is used to smooth the signal, after which the points that have less than half the maximum intensity measured are removed from the dataset. Then, a curve described by the LogNormal model is fitted to the processed signal. During the fitting, a quadratic error function is used. Finally, the peak wavelength present in the signal is extracted from the parameters describing the fitted curve. The next step in the compensation algorithm is to use the calibration curve to calculate the displacement between the LHN and the surface S. Then, a finite/infinite history filter, such as unnormalized fixed gain gradient, moving average, or moving median, is used to estimate the new position of the linear stage needed to achieve the desired value of the stand-off parameter. Optionally, a PI controller could also be used to further improve the results. After that, a verification is made that the new position is within the operating range of the compensation system and that the displacement is greater than the assumed field of insensitivity (FoI), which is used to ignore negligible position deviations in order to limit unnecessary movement of the LHN. If all conditions are met, a command is sent to the controller initiating movement to the designated position.

It should also be mentioned that the spectral signal acquisition time depends on the surface properties. Together with the data processing time, they determine the response time of the system, i.e., the time delay. For the reference material, the response time was 14.7 ms, while for the steel substrate it was up to 64.2 ms.

### 2.3. Verification of Laser Head Displacement Using Digital Image Correlation

**Figure 3:**
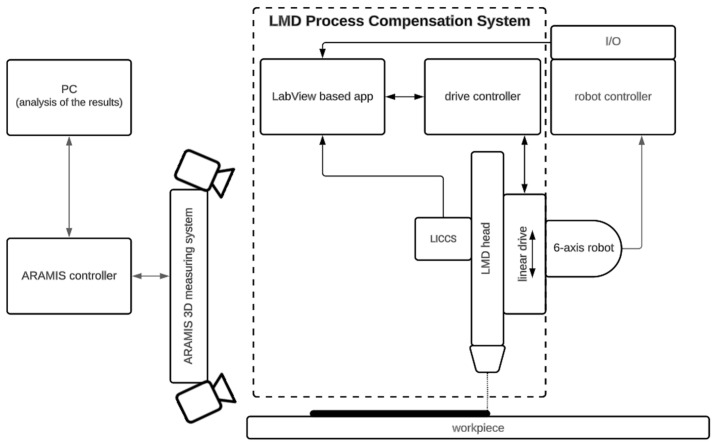
Schematic diagram of the measurement station used to verify the LICCS system for compensation of the stand-off parameter.

Verification of the designed LMD-PCS was performed with the digital image correlation (DIC) system GOM Aramis, which is an external vision-based measuring system that allows for tracking of points in 3D space to measure their displacement. A diagram of the entire measurement system and its implementation is presented in Figure 3.

**Figure 4:**
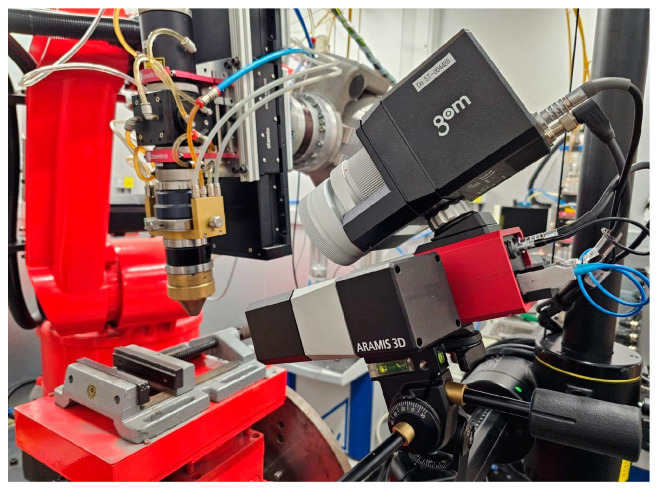
The measurement station used to verify the LICCS system for compensation of the stand-off parameter, i.e., the LMD process station with the Aramis 3D measuring system.

The LMD-PCS implemented for a six-axis industrial robot, as presented in Figure 4, together with the DIC measuring system were used to verify the results. The GOM Aramis DIC system takes measurements based on stereoscopic vision, where the markers are tracked by two cameras, capable of capturing images with a frequency of up to 130 Hz (130 frames per second). The use of the shortest 150 mm beam separating the cameras allowed for the measurement volume of 140 mm × 90 mm × 90 mm.

**Figure 5:**
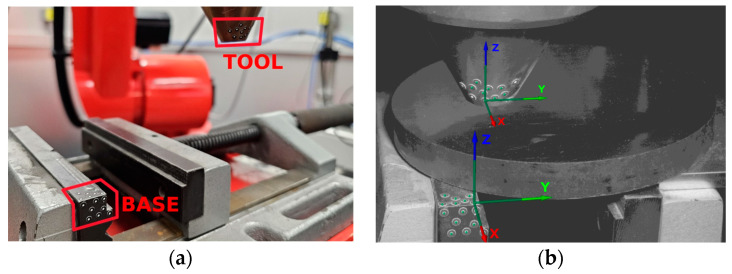
The measurement station with the Aramis 3D measuring system, i.e., markers on the base and the tool used during the DIC analysis: (**a**) Photo of the measurement site; (**b**) view from the measurement application—GOM Correlate Pro.

In this application, two rigid components were defined in the DIC system based on point markers placed on the laser head nozzle tool and the workpiece base (see Figure 5). All of the measurements were performed with rigid body motion compensation (RBMC), where the position of the tool was measured in relation to the static base.

As in the case of this publication, the digital image correlation method has also been used successfully by other researchers in robotic applications. For example, the displacement of the robot tool center point (TCP) was measured by Vocetka et al. [26] to prove that the direction of the approach to the desired position matters for the repeatability of a six-axis industrial robot. Digital image correlation has also been used in mobile robotics as in the case of the analysis of the locomotion of a snake robot designed by Virgala et al. [27], where the movement of the robot was first modeled virtually in MATLAB, and then tested in a real-life scenario using DIC. Digital image correlation can also be used for measuring deformation and has been used for the analysis of the deformation of a wing of a flapping wing aerial vehicle (FWAV) designed by Ariel Perez-Rosado et al. [28], where a correlation between the lift and thrust forces produced by the wings and the biaxial and shear strains was observed on the wings’ surfaces. DIC has also been used with success for verification of repeatable motion of physical mechanisms in medical applications, such as in the case described by Rychlik et al. [29], where a hip joint was tested using a six-axis industrial robot and observed by using the GOM Aramis 3D DIC system.

## 3. Results and Discussion

### 3.1. Effect of Changing the Stand-Off Parameter Values on the Results of the Laser Surfacing Process

The first step of the research involved performing deposition of single clads for different stand-off parameter values. The deposition was repeated five times to perform a statistical analysis of the resultative geometries.

Every clad deposited for a given value of the stand-off parameter was scanned using the laser profiler Keyence LJ-7020 to determine the average values of its height and width. The standard deviations of these geometric quantities were also calculated, considering that these were suitable parameters for the variability of the deposition result.

**Figure 6:**
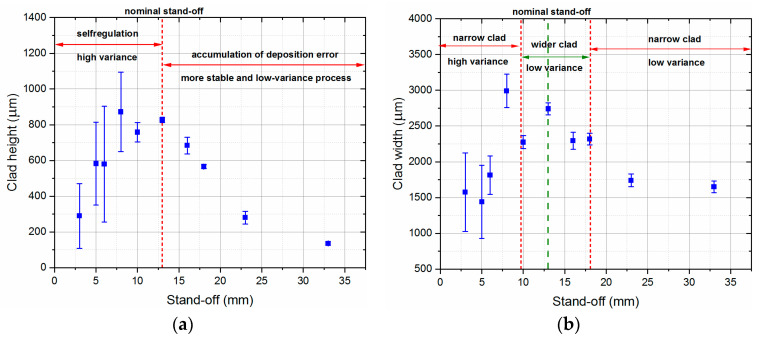
Results of the LMD process carried out with variable stand-off parameter values: (**a**) Clad height; (**b**) clad width.

Figure 6 shows the average values of clad height and width and the variability of these geometrical properties versus the stand-off parameter value. In Figure 6a, where the results of the clad height measurements are presented, two ranges of the stand-off parameter can be distinguished: a self-regulation zone for distances smaller than the nominal stand-off parameter value (13 mm) and a range where an accumulation of material deposition error occurs. In the first case, lower clads were obtained, which should lead to adjustment of the stand-off distance between the nozzle and the deposited structure to the nominal and expected value during the multilayer LMD process. However, for these stand-off distances, there were also significant variations in the height of the clad observed. This means a loss of stability during the LMD process, which is particularly noticeable in the range from 3 to 8 mm. This makes the deposition at stand-off values of less than 10 mm considerably more difficult and can lead to quality defects. In the case of stand-off values exceeding 13 mm, an initial linear decrease in clad height is noticeable with an increase in the stand-off parameter value, which then flattens. Although, the deposition results exhibit low variance and stability for this stand-off parameter range, it is essential to compensate for the accumulation of height error that occurs during multilayer processes.

As shown in Figure 6b, three zones were distinguished, considering the average clad width and its standard deviation. The first and third zones are characterized by a similar average level of the clad width (approximately 1500 µm), but differ in the variations occurring. Nevertheless, both of these zones should be considered unsuitable for the LMD process. The middle zone, between 10 and 16 mm, allows deposition of clad with a width of approximately 2300 µm (16% narrower than for the nominal stand-off parameter value), but the variability of the process is relatively low.

Moreover, the maximal height error of 84% in relation to the clad deposited with the nominal stand-off parameter value is obtained, whereas the maximal width error is 42%. This implies that clad height is more sensitive to changes in the stand-off parameter value than its width. However, both plots indicate that there is a specific range of stand-off parameter values where the resultative clads can have similar geometrical properties and the process remains stable.

The deviating results, which can be seen in Figure 6 for a stand-off parameter value of 8 mm, indicate a relative shift of the focus of the powder stream and the laser beam. Since the value of 13 mm is the working point of the LMD process nozzle, which corresponds to the focus of the powder stream, the laser beam should be considered to have its focus 5 mm higher. This manifests itself in the possibility of obtaining the highest and widest deposit for this distance; however, due to the deposition beyond the powder stream focus, the variance is higher than for the nominal stand-off parameter value of 13 mm. It should be mentioned that this effect is a direct result of the construction of the deposition head, which does not allow a precise adjustment of both foci.

**Figure 7:**
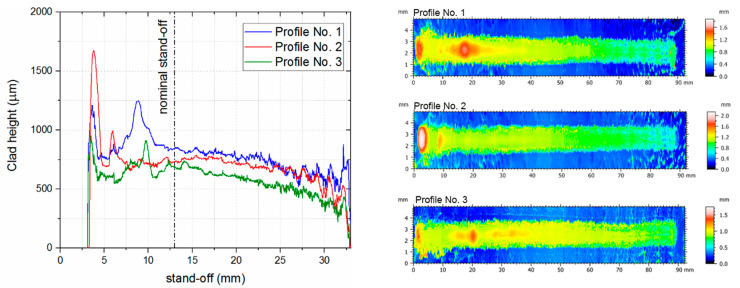
Results of the measurements of clad made with continuous changes in the stand-off parameter value in the range from 3 to 33 mm, over a length of 90 mm: Longitudinal profiles of the clads and color maps showing the height of the resulting clads when the stand-off parameter is continuously changing during the LMD process.

In order to quantitatively characterize this range, an experiment was performed with a continuous change of stand-off distance (three separate trials). The obtained 90 mm long clads were subsequently scanned using an interferometric microscope (Taylor Hobson, Talysurf CCI, Warrenville, IL, USA) to obtain height maps and longitudinal profiles of clads (Figure 7). The analysis of the obtained profiles allowed us to conclude that the stand-off range from 10 to 16 mm should allow for repeatable material deposition during the multilayer LMD process. In other words, both unstable processes (small stand-off values) and superposition of height errors (large stand-off values) should be avoided.

### 3.2. Compensation in an Open-Loop Control System

**Figure 8:**
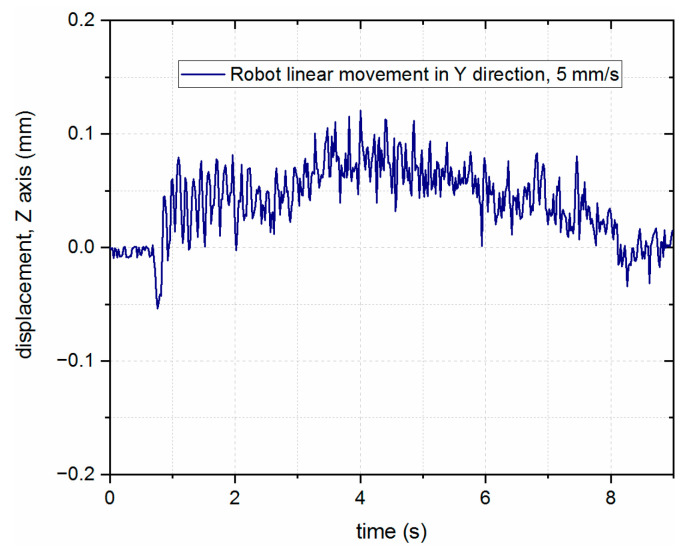
Changes in the head position in the *Z*-axis direction of the robot detected during rectilinear movement in the *Y*-axis direction.

To verify the feasibility of compensating by using the additional axis mounted on the robot end effector, several tests were performed and measured using DIC. First, for reference, the nominal movement of the robot itself was measured during its movement along the surface of the material, which is presented in Figure 8. The Z component of the linear path repeatability of the robot (perpendicular to the surface of the material) was measured in the range of up to 0.2 mm.

Secondly, verification of the proposed external measurement system was carried out to validate the developed system for compensation of the stand-off parameter. For this purpose, a trial movement of the robot was performed so that the TCP of the laser head nozzle moved sequentially along a straight line in the direction of the *Y*-axis of the user-defined coordinate system and along a ramp in the YZ plane with an angle of inclination described by a tangent function of 1/3.

**Figure 9:**
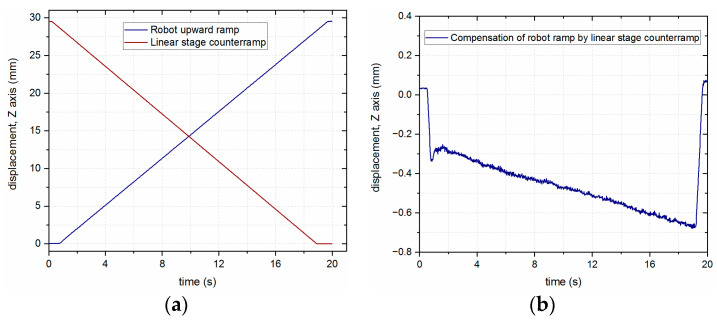
Asynchronous compensation of the laser head nozzle position using an open-loop approach: (**a**) The ramp performed by the robot arm and the counter ramp performed by the linear stage; (**b**) combination of robot and linear stage movements.

Finally, the operation of the system was checked with the open-loop control system, where both the robot and the additional axis moved independently to cancel their movements (see Figure 9a). The resulting displacement in the *Z*-axis when combining these movements together is presented in Figure 9b.

The overall displacement may not have been constant in this case due to slight variations among the reaction time, speed, and acceleration of both the robot and the linear stage. However, it is necessary to specify that the presented mode of operation was not the intended purpose for the designed system, and the described tests were carried out to methodically present the characteristics of the developed solution for the workstation performing the LMD process.

### 3.3. Compensation in a Closed-Loop Control System

To eliminate the issues associated with varying the speed and acceleration between the robot and the linear stage, a closed-loop control system was implemented, where the information from the LICCS sensor was introduced to compensate for the movements of the laser head in relation to the surface of the material. To test different approaches and parameters, the first tests were performed on a reference substrate in the form of a silver mirror, in order to achieve the most accurate results from the LICCS sensor. Once the algorithm was verified on the reference substrate, additional tests were conducted on a steel substrate, resembling the materials used in the industrial application of LMD.

#### 3.3.1. Tests Conducted on the Reference Substrate

**Figure 10:**
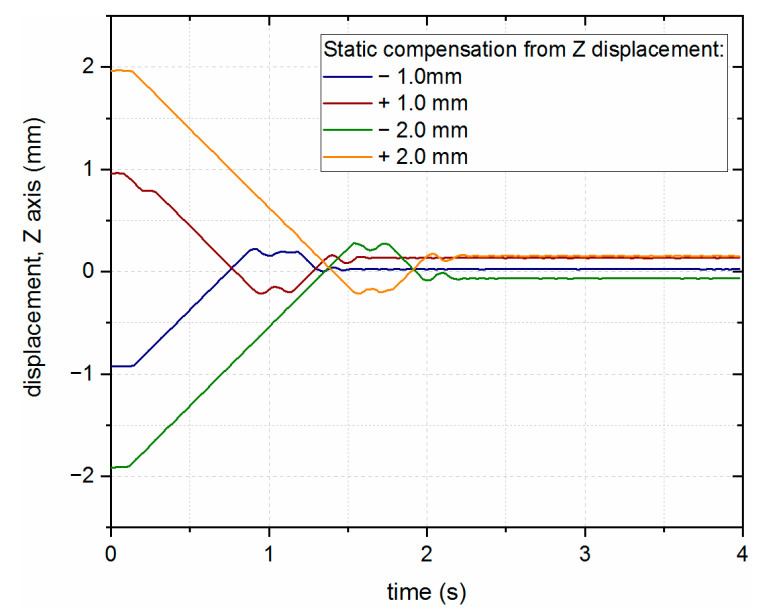
Static LHN position compensation when the working point is incorrectly set within the measurement range of the LICCS system.

First, static compensation was tested, where the position of the laser head was displaced from the desired position by +/−1 mm and +/−2 mm, after which the system moved to the initially specified position. The results presented in Figure 10 show the stabilization of the additional axis around the nominal position of the laser head (zero in the vertical axis). The static error visible in the results may have been caused by the set value of the FoI of the LICCS sensor.

**Figure 11:**
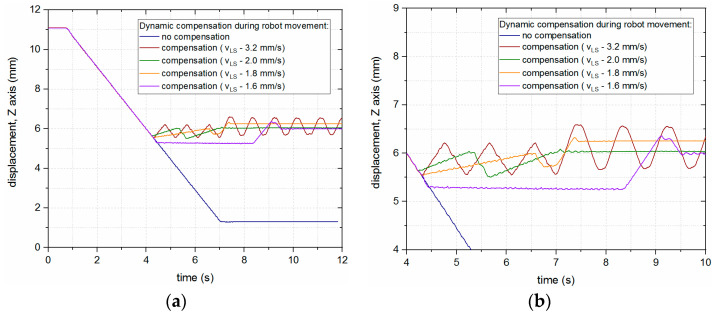
Dynamic LHN position compensation (continuous downward motion of the robot) for different linear stage speed settings: (**a**) Full chart; (**b**) inset.

A test was also performed for dynamic compensation, where the robot was constantly moving the laser head downwards (towards the surface of the material), while the linear stage was compensating for the movement in order to keep a constant distance between the laser head and the surface of the material. With this simple approach, the speed of the linear stage had a significant impact on the behavior of the system, which is presented in Figure 11.

**Figure 12:**
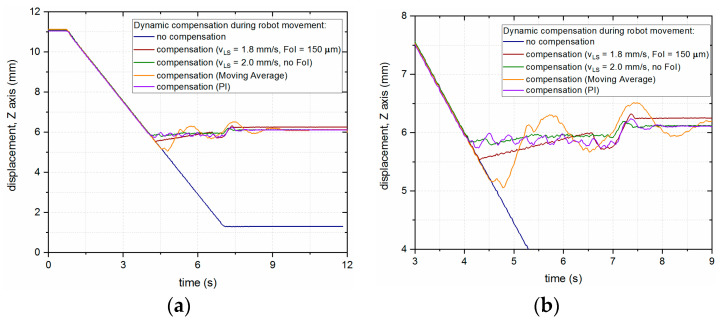
Dynamic LHN position compensation (continuous downward motion of the robot) for different control algorithms for the LICCS system: (**a**) Full chart; (**b**) inset.

As can be seen from the previous figure, the presented approach did not yield good enough results, which prompted the authors to try different control algorithms for compensation of the robot’s vertical motion. The behaviors of the LMD-PCS for different control approaches are presented in Figure 12, where the best results for compensation are achieved with slightly higher speed of the linear stage and no FoI, as well as controlling the system in a closed loop with a PI controller (KP = 0.8 and Ti = 0.02 min).

**Figure 13:**
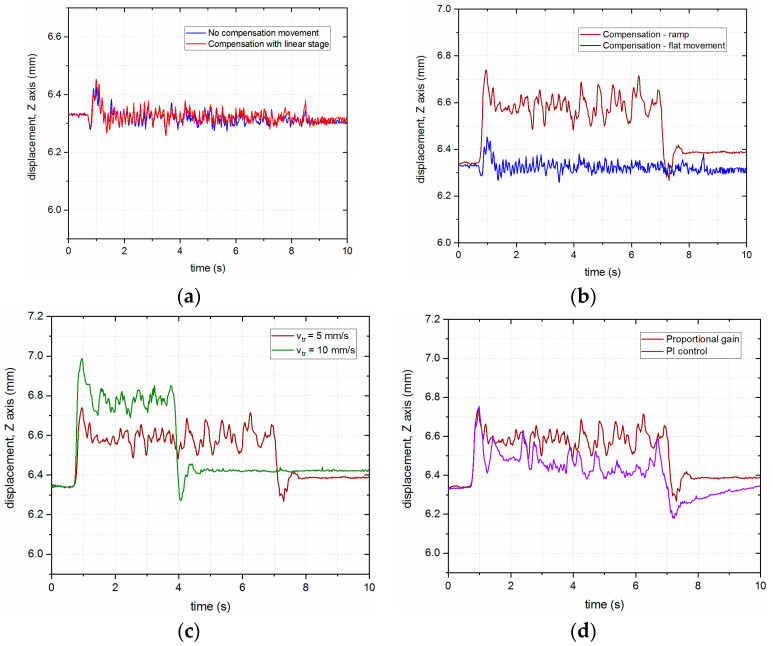
Compensation during linear movement of the robot arm: (**a**) Linear movement along the *Y*-axis, i.e., stability testing; (**b**) comparison of compensation during ramp and flat movement of the robot arm; (**c**) ramps performed with different robot speeds; (**d**) P vs. PI control algorithm.

Additional tests were conducted along the surface of the reference material to investigate how the system behaved with movements resembling the ones performed during the LMD process. Figure 13a presents the movement of the robot along the surface of the material with and without compensation of the linear stage; in both cases, the system behaves in the same way, which means that the feedback loop of the LMD-PCS does not affect the accuracy of the robot negatively.

Subsequently, the robot moved along a ramp above the surface of the material (composition of movement along the *Y*-axis and the *Z*-axis), where the system responded with a delayed reaction to the initial movement and was compensating for the movement based on the previous reading; hence, the steady-state error is visible in Figure 13b. This steady-state error occurs due to the inertia of the system and is dependent on the ratio of the vertical speed of the linear stage and the horizontal speed of the robot, which can be seen in Figure 13c, where the speed of the robot has doubled. Lastly, tests were also performed for different values of the PI controller, which can be seen in Figure 13d.

#### 3.3.2. Tests Conducted on the Steel Substrate

As was mentioned earlier, measurement systems based on CCS perform much worse for surfaces with large differences in roughness, containing irregularities after machining. The accuracy of such systems is highly dependent on the quality of the surface under investigation [30]. This is the reason why, for the uneven surface of the steel substrate in comparison to the reference, operation of the system was tested for different values of the FoI parameter. In all cases, if not stated otherwise, an unnormalized gradient adaptation algorithm with fixed gain was used to improve the results.

**Figure 14:**
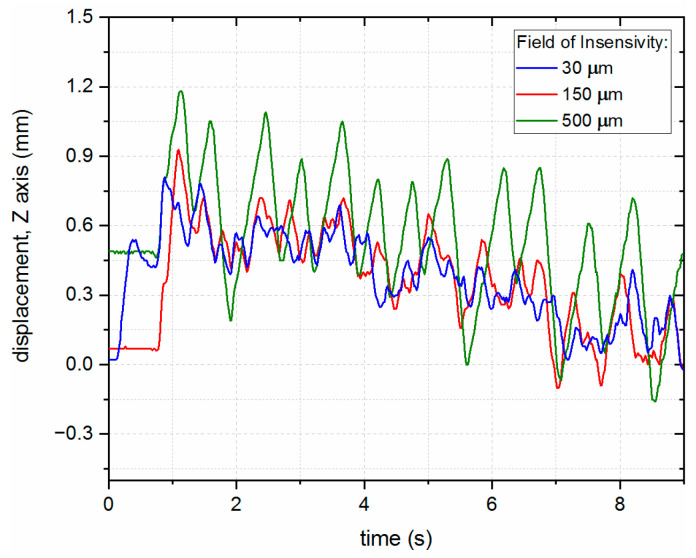
Compensation of the LHN during ramp movement of the robot ramp over the steel substrate with different FoI parameter settings.

The results presented in Figure 14 show that the wider the FoI, the higher the displacement of the end effector, which is undesirable, suggesting that this parameter should be minimized.

**Figure 15:**
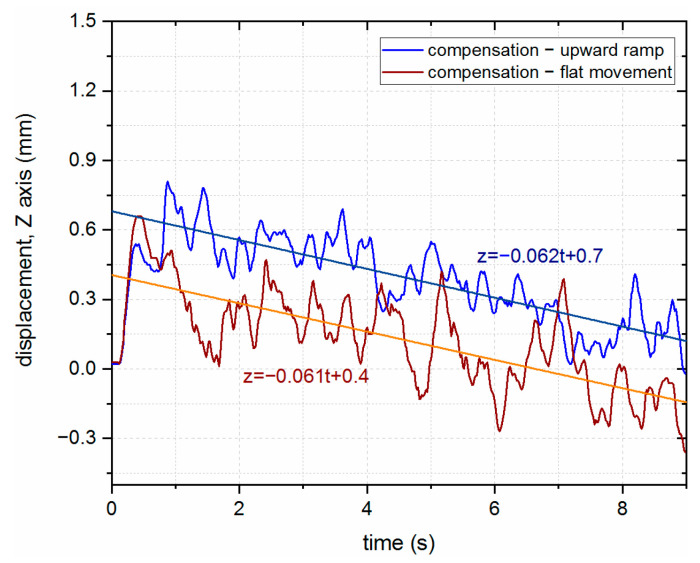
Comparison of the LHN compensation results with flat and ramp movement for the steel substrate with linear fit proposed.

Even though the surface of the steel substrate is rougher, the movement of the laser head with variations was compensated for by using the LMD-PCS; movement of the robot along both the surface with and without the vertical component was characterized by a trend line with nearly the same slope coefficient, which suggested that the ramp was properly compensated for by the control system. The vertical displacement of the trend line presents the steady-state error caused by the inertia of the system (see Figure 15).

**Figure 16:**
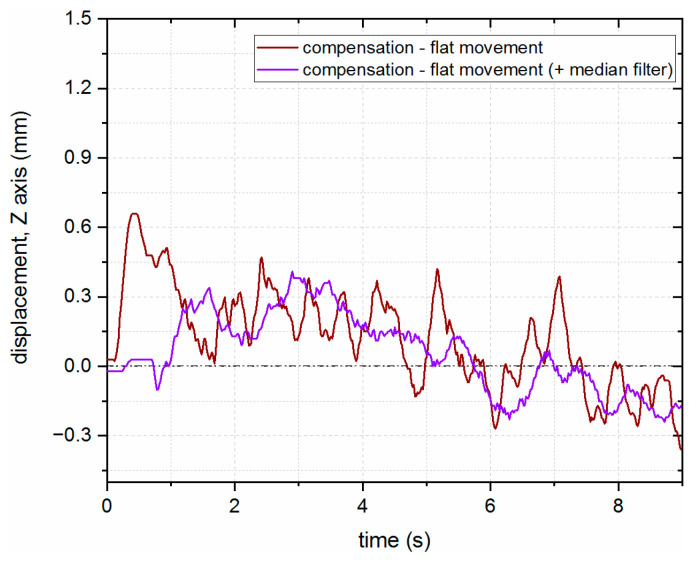
Comparison of the LHN compensation results with flat movement for the steel substrate with an additional median filter implemented during the signal processing.

For better results on steel substrates, where an uneven, rough surface can cause outliers to appear, a median filter was applied to the signal from the LICCS sensor, which improved the behavior of the system and reduced the overall displacement from the nominal distance to the surface, as presented in Figure 16.

## 4. Conclusions

In this preliminary study on the effects of the stand-off parameter on the performance of the LMD process, a zone of self-regulation of material application was identified, while indicating that the process may lose stability for values of the stand-off parameter smaller than the nominal value. The range of acceptable changes in the stand-off parameter for the AA13 nozzle of the COAXpowerline system was also specified as equal to +/−3 mm from the nominal value.

The LMD-PCS system was built, which consisted of the developed LICCS measurement system and a linear stage actuator, managed by a stand-alone LabView-based application. Various algorithms were also verified to enhance the control of the linear stage position based on the reading from the LICCS measurement system. Finally, it was concluded that, for static and dynamic compensation during the robot approach movement, FoI should not be used, and the speed of the linear stage should not exceed 125% of the speed of the robot, so that the system does not fall into vibration. However, in the case of flat movement over the steel substrate, the FoI parameter can be used but its value should be minimal. Application on the surface of the engineering material also required additional filtering, with the best results achieved for a combination of a finite history algorithm (median filter) and an infinite history algorithm (unnormalized gradient adaptation algorithm).

The validation tests showed that, in the case of the reference substrate (silver mirror), the total error of the LMD-PCS system was mainly caused by the delay between the implementation of the correction motion by the linear stage and the acquisition of the spectrum by the LICCS system. For the analyzed movement conditions, its value can be estimated to be 0.3 mm. A similar error was observed for the steel substrate.

The system provides significant advantages to the LMD process, allowing for universal integration on any type of manipulator, where the accuracy of the LICCS (35 μm and 90 μm for silver mirror and low alloy steel substrate, respectively) combined with the accuracy of the linear stage (0.4 μm) result in an accuracy of the entire system greater than the movement accuracy of the manipulator itself (200 μm for REIS RV 60-40). While in the current form the system requires calibration of the sensor to each type of material, it can do so automatically, thus, eliminating the need for a specialist. Although the LICCS can be set up in advance to measure the distance ahead of the process to respond, its main advantage is its ability to be implemented coaxially with the laser beam. The ability for coaxial measurement within the diameter of the surfacing nozzle and the full flexibility and modularity/customization of this solution should be considered an advantage over systems using triangulation measurement and laser distance sensors. The measurement system is moved away from the process area, so it is not exposed to damage, and is just an additional module of the optical system of the laser head. However, it is necessary to optimize the system in terms of measurement signal intensity, measurement range, and accuracy. In addition, the influence of high roughness on the measurement result must be considered a disadvantage of this solution.

The focus of further research on the developed system is its application under conditions of performing the LMD process. This requires the use of a broadband light source with sufficient optical power so that readings will not be corrupted by residual radiation from the process.

## Figures and Tables

**Table 1:** Parameters of the LMD process during preliminary studies on the impact of stand-off parameter values on the clad geometry.

Parameter	Abbreviation	Value
Laser spot diameter	d_las_	1.5 mm
Laser beam power	P_las_	600 W
Travel speed	v_tr_	5 mm/s
Powder feed rate	f_pwd_	17.5 g/min
Carrier gas flow rate	Q_cr_	2 L/min
Shielding gas flow rate	Q_sh_	8 L/min

## Data Availability

The data acquired for verification of LMD-PCS are available from the corresponding authors on reasonable request.
